# Supplementary material for: A sulfur-aromatic gate latch is essential for opening of the Orai1 channel pore
Source: eLife. 2020 Oct 30;9:e60751. doi: 10.7554/eLife.60751 (PMC7679135; doi:10.7554/eLife.60751)
Supplement: Figure 3—source data 1. [file elife-60751-fig3-data1.docx]

Figure 3 Numerical Data

**Figure 3D**

| **Mutant** | **Fold Change** | **SEM** | **N** | **T-test p-value (vs. WT)** |
| --- | --- | --- | --- | --- |
| WT | 0.93 | 0.02 | 4 | --- |
| F99C/M101C | 10.5 | 1.7 | 7 | 0.0012 |
| F99C/M101C + STIM1 | 2.1 | 0.91 | 5 | 0.26 |

**Figure 3G**

20 mM Ca^2+^ external solution

| **Concentration (µM)** | **Fold Change** | **SEM** | **N** |
| --- | --- | --- | --- |
| 0.3 | 1.2 | 0.16 | 4 |
| 1 | 2.9 | 0.17 | 5 |
| 5 | 10.5 | 1.7 | 7 |
| 10 | 27.7 | 2.1 | 4 |
| 50 | 40.9 | 4.6 | 11 |
| 500 | 152.9 | 26.8 | 7 |

110 mM Ca^2+^ external solution

| **Concentration (µM)** | **Fold Change** | **SEM** | **N** | **T-test p-value (vs. 20 mM)** |
| --- | --- | --- | --- | --- |
| 0.3 | 1.2 | 0.05 | 4 | 0.48 |
| 1 | 2.0 | 0.39 | 5 | 0.050 |
| 5 | 3.9 | 0.69 | 5 | 0.0034 |
| 10 | 12.3 | 3.1 | 6 | 0.0017 |
| 50 | 17.4 | 5.1 | 7 | 0.0021 |
| 500 | 82.0 | 24.3 | 5 | 0.040 |
