# Supplementary material for: A sulfur-aromatic gate latch is essential for opening of the Orai1 channel pore
Source: eLife. 2020 Oct 30;9:e60751. doi: 10.7554/eLife.60751 (PMC7679135; doi:10.7554/eLife.60751)
Supplement: Figure 4—source data 1. [file elife-60751-fig4-data1.docx]

Figure 4 Numerical Data

**Figure 4C**

| **Mutant** | **% Block** | **SEM** | **N** | **T-test p-value (vs. WT)** |
| --- | --- | --- | --- | --- |
| WT | 7.6 | 2.4 | 4 | --- |
| M101C/F187C | 83 | 2.7 | 5 | 1.5*10^-7^ |
| M101C/F187C + STIM1 | 77 | 4.4 | 4 | 6.0*10^-5^ |
| F187C | 1.5 | 2.7 | 5 | 0.14 |
| M101A/F187C | 6.4 | 3.0 | 6 | 0.75 |
| M101C/H134S | 8.5 | 3.3 | 7 | 0.84 |

**Figure 4F**

20 mM Ca^2+^ external solution

| **Concentration (µM)** | **% Block** | **SEM** | **N** |
| --- | --- | --- | --- |
| 0.01 | 7.3 | 1.7 | 7 |
| 0.05 | 20 | 4.8 | 5 |
| 0.1 | 35 | 3.4 | 4 |
| 0.3 | 60 | 4.1 | 7 |
| 1 | 71 | 2.7 | 5 |
| 5 | 78 | 2.1 | 8 |
| 10 | 76 | 1.1 | 5 |
| 50 | 81 | 3.6 | 4 |

110 mM Ca^2+^ external solution

| **Concentration (µM)** | **% Block** | **SEM** | **N** | **T-test p-value (vs. 20 mM)** |
| --- | --- | --- | --- | --- |
| 0.05 | 11 | 2.9 | 5 | 0.15 |
| 0.1 | 28 | 3.6 | 5 | 0.21 |
| 0.3 | 46 | 3.4 | 7 | 0.02 |
| 1 | 66 | 1.5 | 4 | 0.13 |
| 5 | 77 | 1.5 | 7 | 0.62 |
| 10 | 75 | 1.8 | 6 | 0.51 |
| 50 | 75 | 1.4 | 4 | 0.20 |

**Fit parameters:**

20 mM Ca^2+^ 110 mM Ca^2+^

Max = 0.79 ± 0.02 Max = 0.76 ± 0.02

n = 1.17 ± 0.13 n= 1.12 ± 0.13

t_1/2_ = 0.12 ± 0.01 t_1/2_ = 0.19 ± 0.02
