# Supplementary material for: A sulfur-aromatic gate latch is essential for opening of the Orai1 channel pore
Source: eLife. 2020 Oct 30;9:e60751. doi: 10.7554/eLife.60751 (PMC7679135; doi:10.7554/eLife.60751)
Supplement: Figure 5—source data 1. [file elife-60751-fig5-data1.docx]

Figure 5 Numerical Data

**Figure 5A**

| **Mutant** | **Current Amplitude (pA/pF)** | **SEM** | **N** | **T-test p-value (vs. WT)** |
| --- | --- | --- | --- | --- |
| WT | -33.5 | 4.1 | 9 | --- |
| M101G | -3.1 | 0.89 | 5 | 1.1*10^-4^ |
| M101A | -0.62 | 0.24 | 6 | 7.4*10^-5^ |
| M101S | -0.64 | 0.15 | 5 | 7.5*10^-5^ |
| M101T | -0.51 | 0.12 | 5 | 7.3*10^-5^ |
| M101C | -0.51 | 0.12 | 4 | 7.3*10^-5^ |
| M101V | -2.2 | 0.67 | 5 | 9.4*10^-5^ |
| M101L | -0.38 | 0.13 | 4 | 7.1*10^-5^ |
| M101I | -1.2 | 0.33 | 5 | 8.3*10^-5^ |
| M101F | -41.2 | 13.9 | 4 | 0.48 |

**Figure 5C**

| **Mutant** | **Current Amplitude (pA/pF)** | **SEM** | **N** | **T-test p-value (vs. WT)** |
| --- | --- | --- | --- | --- |
| WT | -0.19 | 0.02 | 4 | --- |
| M101F | -7.7 | 1.9 | 5 | 0.016 |

**Figure 5 Supplement 1B**

| **Mutant** | **E-FRET** | **SEM** | **N** |
| --- | --- | --- | --- |
| WT | 0.31 | 0.01 | 51 |
| M101T | 0.30 | 0.01 | 72 |
| M101V | 0.28 | 0.01 | 70 |
| M101L | 0.36 | 0.01 | 65 |

**Figure 5 Supplement 2A**

| **Mutant** | **Current Amplitude (pA/pF)** | **SEM** | **N** | **T-test p-value (vs. H134S)** |
| --- | --- | --- | --- | --- |
| H134S | -29.7 | 3.4 | 6 | --- |
| M101C/H134S | -5.8 | 1.2 | 6 | 0.00047 |
| M101L/H134S | -7.7 | 0.69 | 5 | 0.0011 |

**Figure 5 Supplement 2B**

| **Mutant** | **Current Amplitude (pA/pF)** | **SEM** | **N** | **T-test p-value (vs. V102C)** |
| --- | --- | --- | --- | --- |
| V102C | -26.6 | 7.4 | 6 | --- |
| M101L/V102C | -2.4 | 0.29 | 6 | 0.022 |

**Figure 5 Supplement 2C**

| **Mutant** | **Current Amplitude (pA/pF)** | **SEM** | **N** | **T-test p-value (vs. H134W)** |
| --- | --- | --- | --- | --- |
| H134W | -0.17 | 0.08 | 5 | --- |
| H134W + STIM1 | -0.84 | 0.37 | 4 | 0.078 |
| M101F/H134W | -6.8 | 2.1 | 6 | 0.025 |

**Figure 5 Supplement 3**

| **Mutant** | **Current Amplitude (pA/pF)** | **SEM** | **N** | **T-test p-value (vs. WT)** |
| --- | --- | --- | --- | --- |
| WT | -0.17 | 0.08 | 5 | --- |
| F187G | -2.8 | 0.15 | 4 | 0.00033 |
| F187A | -4.0 | 0.90 | 6 | 0.013 |
| F187S | -7.2 | 2.6 | 4 | 0.036 |
| F187C | -7.4 | 2.3 | 6 | 0.026 |
| F187L | -0.32 | 0.06 | 4 | 0.11 |
| F187Y | -0.26 | 0.09 | 5 | 0.46 |
| F187W | -0.30 | 0.14 | 5 | 0.47 |
